# Reasoning to Justify Eating Animals Varies With Age

**DOI:** 10.1111/cdev.14217

**Published:** 2025-02-01

**Authors:** Luke McGuire, Tina Bagus, Alexander G. Carter, Emma Fry, Nadira S. Faber

**Affiliations:** ^1^ University of Exeter Exeter UK; ^2^ DIPF Leibniz Institute for Research and Information in Education Frankfurt Germany; ^3^ University of Bremen Bremen Germany; ^4^ University of Oxford Oxford UK

**Keywords:** human–animal intergroup relations, meat eating, reasoning

## Abstract

The present study examined the justifications used by children, adolescents, and adults to justify eating animals. Children (*n* = 100, *M* ^age^ = 9.82, SD = 0.77, female *n* = 49) as compared to adolescents (*n* = 76, *M* ^age^ = 14.0, SD = 1.62, female *n* = 36) and adults (*n* = 113, *M* ^age^ = 44.1, SD = 14.4, female *n* = 54) were more ambivalent or opposed to eating animals, and they showed a distinct reasoning pattern. Children relied less on arguments about meat eating being natural or with to humane slaughter practices. These findings align with recent theoretical perspectives that reasoning may be used to counter cognitive dissonance arising from knowledge of food production systems.

Eating a diet that includes animal products has consequences for the climate crisis (Clark et al. 2020), related social justice problems (e.g., the disproportionate effects of climate change on low‐ and middle‐income countries; Levy and Patz 2015), for individual health (Wolk 2017), and for animal wellbeing (Knowles et al. 2008). Humans' dietary choices have long‐term consequences, contributing to individual health (Papier et al. 2023) and planetary health (Clark et al. 2020). Currently, approximately 7% of adults in the United Kingdom are eating a vegetarian or vegan diet (YouGov 2025). Efforts to convince adults to take up plant‐based diets (see Kwasny, Dobernig, and Riefler 2022 for a review) have had mixed success. This is, in part, due to the powerful psychological processes that support meat eating in adulthood, including reasoning (Bastian and Loughnan 2017; Loughnan, Bastian, and Haslam 2014). Through reasoning—the justifications provided when asked why it is okay to eat animals—adults can balance their concerns for animal welfare and continue eating a diet that includes animal products.

In comparison to adults, we know less about how children in Western, industrialized countries like the United Kingdom reason about eating animals. Less still is known about this process in adolescence where research has yet to examine moral judgments about eating animals. In addition, research to date (e.g., McGuire et al. 2023) has most often pitted “opponents” against “proponents” of meat eating without focusing on more ambivalent reasoners who neither strongly advocate for nor clearly oppose eating animals. In the present paper, we examined the role of age in evaluations and reasoning about eating animals among children, adolescents, and adults in the United Kingdom. Crucially, we investigated whether age was related to being a member of opponent, proponent, or ambivalent groups, and in turn the types of reasoning associated with these groups.

## 1 Moral Concern for Animals and Evaluation of Meat Eating Across the Life Span

Research has documented the sophisticated psychological processes that adults use to justify eating animals while simultaneously stating an opposition to harming animals. For example, adults diminish the intelligence and sentience they attribute to farmed animals (Loughnan, Haslam, and Bastian 2010), create hierarchies of concern placing some animals above others (Caviola et al. 2021), and engage in reasoning that justifies these hierarchies as “natural” (Piazza et al. 2015). In contrast, recent research has documented that children instead prioritize animal lives and support animal welfare arguments.

For example, one study demonstrated that in moral dilemmas, 5‐ to 9‐year‐old children in the United States were just as likely to save the lives of humans, dogs, and pigs, compared to adults who would save one human life over 100 pigs (Wilks et al. 2021), a finding that has been recently replicated in Poland (Paruzel‐Czachura et al. 2024). Similarly, 9‐ to 11‐year‐old children in the United Kingdom have been shown to report that it is less morally acceptable to eat animals, to be more likely to categorize farmed animals as “pets” rather than “food” and to report that pigs ought to be treated better than adults do (McGuire, Palmer, and Faber 2023). In addition, in this study, children also scored lower than adults on a standardized measure of “speciesism,” that is, the belief that the moral value of an individual is determined solely by its species membership (Caviola, Everett, and Faber 2019; Singer 1975, 2023).

These findings appear in part to be related to animals' status as *food*. For example, 6‐ to 10‐year‐old children in the United Kingdom are less likely to use edibility as a criterion for estimating the moral worth of an animal (Henseler Kozachenko and Piazza 2021). Children are also less strategic in their use of information about other characteristics such as intelligence, whereas adults take such information into account when determining the moral concern that ought to be granted to an animal (Henseler Kozachenko and Piazza 2023). Despite this evidence that suggests children prioritize animal lives and welfare more than adults do, most children eat an omnivorous diet including at least some animal products. Adults use reasoning as one means to justify eating animals (Piazza et al. 2015). One possibility that has so far been underexplored is that the early roots of this reasoning emerge in childhood and adolescence alongside competing concerns for animal welfare. In the present paper, we apply theories of moral development and reasoning to better understand the types of reasoning adults have shown in existing work and the kinds of reasoning endorsed by children.

## 2 Social Domain Theory and Reasoning

Social domain theory (Smetana, Jambon, and Ball 2014; Turiel 1983) is a theory of moral judgment and reasoning that proposes social decisions are made using three domains of knowledge. These include the *moral* domain (issues involving harm, welfare, justice, fairness), the *social‐conventional* domain (issues involving norms, conventions, rules), and the *personal* domain (issues involving personal choice and autonomy). Unlike previous stage‐based models of moral judgment development which framed moral reasoning as a linear developmental process, social domain theory argues that children and adolescents can use and coordinate these three domains of knowledge simultaneously from a young age.

This is particularly important for the present work as each domain makes a relevant contribution to arguments about whether humans ought to eat animals. For example, arguments against eating animals often rely on moral domain appeals to animal rights and welfare, while arguments in favor of eating animals appeal to the social‐conventional domain that eating meat is highly normative. Research from the social domain theory perspective has documented increasingly sophisticated use of multiple competing domain issues with age (Richardson, Mulvey, and Killen 2012) that align with existing work on reasoning about eating animals (Hussar and Harris 2010; McGuire et al. 2023). Some of the reasoning categories most often used by adults to justify eating animals map onto these domain concerns. Adults have been shown to use the so‐called 4Ns, that is, to argue that eating meat is *natural* (“our ancestors have always done it,” “we are designed to eat meat”), *necessary* (“we need protein from meat to survive”), *normal* (“everyone else around me is doing it”), and *nice* (“I like the taste of meat”; Joy 2011; Piazza et al. 2015). Such studies document that the social‐conventional (normal) and personal (nice) domains are highly salient when adults are asked why it is okay to eat animals.

In terms of burgeoning work on reasoning among children, studies have documented the emerging concerns that are relevant to decision making in this area. For example, one paper surveyed independent vegetarian children aged 6–10 years old in the United States (i.e., children who had stopped eating meat independent of their omnivorous parents) who reported that they had changed their diet out of concern for animal welfare (Hussar and Harris 2010). Interestingly, the same children reported that if someone had *not* made a moral commitment to become a vegetarian, then it would be acceptable for them to eat animals. This finding indicates an early awareness that some young children can begin to understand the complex motivations for eating animals. That is, these independent vegetarian children can coordinate their own moral domain concerns with personal domain appeals to personal choice. Relatedly, a recent study from a social domain theory perspective documented that 9‐ to 11‐year‐old children in the United Kingdom, compared to adults, made greater reference to animal welfare arguments (i.e., relied on the moral domain), compared to adults who made use of “natural” and “necessary” arguments (McGuire et al. 2023).

To date, studies examining children's and adults' reasoning about eating animals have identified that there are concerns relevant to the moral, social‐conventional, *and* personal domains (McGuire et al. 2023; Piazza et al. 2015). Crucially, these studies have shown that the domains of knowledge that are salient depend on the position of the individual. Opponents of eating meat rely on moral domain concerns (although even in childhood these opponents can recognize that diet involves the personal domain), and proponents make appeals to conventions around food. There are two key areas that these studies have yet to focus on. First, much less is known about how *adolescents* reason about eating animals. This is of particular interest as other research driven by social domain theory has documented the increasing importance of norms (McGuire et al. 2019) and personal autonomy (Smetana and Asquith 1994) in this age range suggesting that this may be where more “adult” thinking on the topic of eating animals may emerge with reference to the 4 N's and a focus on dietary autonomy over animal welfare. Second, previous studies have pitched proponents against opponents of eating animals with less focus paid to individuals who are more “on the fence” about whether it is morally acceptable to eat animals. Given research and theory have documented the conflict that is apparent in eating animals for many people (Bastian and Loughnan 2017; Loughnan, Bastian, and Haslam 2014), it is an important next step in this area to directly focus on the reasoning of this group who are not strongly in favor of *or* opposed to eating animals. Existing work has shown that some adults reference the ethics of meat production to justify meat eating, which may be one way in which ambivalent meat‐eaters balance their existing diet and their concern for animal lives (McGuire et al. 2023).

An additional important element of reasoning regarding eating animals that has not yet been studied among adults, adolescents, or children is the possibility that humans reason about eating animals by using *multiple* types of reasoning at once. For example, one may argue that while it is wrong to eat animals due to the harm involved, we must carry on doing so because eating meat is a normal part of our day‐to‐day lives *and* that there are less harmful ways to rear and kill animals that make this practice more justifiable. Using such arguments directly reflects the cognitive dissonance that many people experience when thinking about the conflict between their concern for animal lives and their dietary practices (Rothgerber 2020b; Rothgerber and Rosenfeld 2021). In the present study, for the first time, we examine this form of “dual” reasoning (where two potentially conflicting arguments are used by the same individual) as well as “single” argument reasoning (where only one argument is used) to examine explicit examples of the reasoning that may result from this dissonance. Due to the exploratory nature of this aim, we did not have expectations for how the use of such reasoning may differ based on participant age.

## 3 The Present Study

Eating animals can trigger psychological conflict between humans' reluctance to harm animals and the knowledge that omnivorous diets involve a tacit endorsement of harm to animals. So far, little work has examined how children and adolescents reason about decisions to eat animals. In the present study, we extended existing work (McGuire et al. 2023; Piazza et al. 2015) by exploring children's, adolescents', and adults' evaluations of eating animals, moving beyond a binary “for or against” categorization to instead examine the reasoning of proponents, opponents, and ambivalent groups at different ages. In addition, recognizing that there is a lack of work that has examined reasoning arguments that include more than one form of argument (i.e., single versus dual arguments), we aimed to examine these forms of argument in more detail across different evaluation groups. We note that in this study, our focus is on children aged 9–11 years in the United Kingdom and use “children” as a shorthand for this group throughout the rest of the paper. Given the relative paucity of evidence in this area and exploratory nature of the study, we formulated the following open research questions (RQs).

Research Question 1: First, we asked whether age was related to being a member of the opponent, proponent, or ambivalent evaluation groups regarding eating animals.

Research Question 2: Second, we asked whether scores on a standardized speciesism scale were related to evaluation group and age group.

Research Question 3: Third, we asked whether the likelihood of using different reasoning categories depended on evaluation group and age.

Research Question 4: Finally, we asked whether the likelihood of using different reasoning categories depended on argument type (single vs. dual argument).

## 2 Method

### 2.1 Open Science

The sample size, materials, and analysis plan of the current study were preregistered on AsPredicted.org (https://aspredicted.org/blind.php?x=HLM_4RL), but the focus on single versus dual arguments was derived post hoc from a bottom‐up read of the data and should thus be considered exploratory rather than confirmatory. Materials, data, and analysis can be found on the Open Science Framework (https://osf.io/uzf2y/).

### 2.2 Participants

The final sample for analysis included 289 participants from 9 to 78 years old, living in the United Kingdom, divided into groups of children (*n* = 100, *M* age = 9.82, SD = 0.77, minimum age = 9 years old, maximum age = 11 years old, female *n* = 49, male *n* = 51), adolescents (*n* = 76, *M* age = 14.0, SD = 1.62, minimum age = 12 years old, maximum age = 17 years old, female *n* = 36, male *n* = 40), and adults (*n* = 113, *M* age = 44.1, SD = 14.4, minimum age = 19 years old, maximum age = 78 years old, female *n* = 54, male *n* = 59). In this study, we chose to include 12‐year‐olds in the adolescent group as these children attended secondary school/high school in the United Kingdom and had therefore undergone an important transition to an adolescent context. As such, we expected these 12‐year‐olds to differ from our 11‐year‐olds who were still in primary school. Participants who identified as an other gender (children *n* = 2, adolescents *n* = 2, adults *n* = 1) or did not report their gender (children *n* = 2, adolescents *n* = 4) were not included in the analyses due to the inclusion of gender as a covariate in the models described below.

Participants identified their race/ethnicity as follows: White British (*n* = 269; 94.1% of sample), Black British (*n =* 4, 1.4%), Bengali British (*n* = 3, 1%), Chinese British (*n* = 1, 0.3%), Indian British (*n* = 1, 0.3%), Pakistani British (*n* = 3, 1%), and Mixed Race/Dual Heritage (*n* = 5, 1.7%). Two‐hundred and nineteen participants reported that they were not religious (76.6%), while the remainder of the sample included Christian (*n* = 54, 18.9%), Jewish (*n* = 6, 2.1%), Muslim (*n* = 2, 0.7%), Sikh (*n* = 2, 0.7%), Buddhist (*n* = 2, 0.7%), and other religious groups (*n* = 1, 0.3%). In terms of diet, the sample included omnivores (*n* = 149, 73.8%), flexitarians (*n* = 18, 8.9%), vegetarians (*n* = 11, 5.4%), vegans (*n* = 9, 4.5%), pescatarians (*n* = 4, 2%), and other dietary groups (*n* = 11, 5.2%). Most children did not report their diet (children omnivores *n* = 11, “other” *n* = 2, did not report *n* = 87). Adolescents were primarily omnivores (*n* = 67, vegetarian *n* = 1, vegan *n* = 1, flexitarian *n* = 1, pescatarian *n* = 1, other *n* = 1, did not report *n* = 4). Adults were also primarily omnivores (*n* = 71, vegetarian *n* = 10, vegan *n* = 8, flexitarian *n* = 17, pescatarian *n* = 3, other *n* = 4). Two‐hundred and twenty‐eight participants (78.9%) reported that they lived with pets or animals at home, while the remaining 61 participants (21.1%) did not.

### 2.3 Procedure and Materials

Children and adolescents completed a survey on Qualtrics in a school classroom setting. All children and adolescents received consent from their parents/guardians to participate and gave their own assent to participate on the day of testing. Adult participants registered to take part via Prolific Academic and gave informed consent before completing the survey on Qualtrics. Data collection took place between April and September 2022. The survey also included animal treatment questions and a pilot version of a food systems knowledge task. The full survey can be found on OSF. Since these tasks are not the focus of interest, they will not be discussed in the current paper.

#### 2.3.1 Speciesism Scale

The speciesism scale (adapted from Caviola, Everett, and Faber (2019) and previously used with children in McGuire, Palmer, and Faber (2023)) included six items (e.g., “Morally, animals always count for less than humans”). Participants were asked to indicate their agreement with each item on a seven‐point Likert scale (1 = *strongly disagree*, 7 = *strongly agree*; α = 0.71).

#### 2.3.2 Evaluation of Eating Animals

Participants were first asked whether it was okay or not okay to eat animals. Second, they were asked to elaborate on a six‐point Likert scale (1 = *really not okay*, 6 = *really okay*) in response to the questions “How okay or not okay is it to eat animals?” Lastly, they were asked “why is it okay or not okay to eat animals?” (open‐ended).

### 2.4 Data Coding and Analysis Plan

We coded open‐ended responses using a coding framework (see Table 1 for definitions and category examples) developed deductively using social domain theory (Smetana, Jambon, and Ball 2014; Turiel 1983) and complemented by inductive category building based on the given answers and recent work in this area (McGuire et al. 2023). The *natural*, *necessary*, and *normal* categories were derived from the 4Ns, with *normal* aligning with the social‐conventional domain. The *animal welfare* and *sustainability* categories were derived from the moral domain. The *personal choice* and *nice* categories are derived from the personal domain. Additionally, a mixed domain category for *humane slaughter* was created, including both social‐conventional and moral concerns. Finally, responses that did not fit in these categories were coded as *other*. The reliability of the reasoning coding was assessed by two independent researchers coding overlapping 23% of the responses. Cohen's κ = 0.96 indicated strong agreement between the two coders. In our analyses, we took categories into account that were used by at least 10% of participants. Therefore, the main categories in the evaluation of eating animals reasoning analysis were *natural* (21%), *necessary* (25%), *normal* (10%), *nice* (12%), *humane slaughter* (12%), and *animal welfar*e (27%). *Other* met the 10% mark, but as this category included various topics, it was not considered in the following analyses.

**TABLE 1:** Reasoning coding framework with definitions and examples.

Category	Definition	Examples
Natural	Positive or negative appeals to biology, biological hierarchy, natural selection, human evolution, or the naturalness of eating meat	“It is natural for humans to eat meat”; “Humans are carnivores”; “Humans were meant to have dominion over animals”
Necessary	Positive or negative appeals to the necessity of meat for survival, strength, development, health, animal population control, or economic stability	“Humans need meat to survive”; “Our bodies need the protein”; “Meat provides good nutrients”; “Protein is a necessary part of our diet”
Normal	Positive or negative appeals to dominant societal norms, normative behavior, historical human behavior, or socially constructed food pyramids	“Society says it's okay”; “I was raised eating meat”; “Meat is culturally accepted”; “A lot of other people eat meat”
Nice	Positive or negative appeals to the tastiness of meat, or that it is fulfilling or satisfying	“It tastes good”; “It's delicious”
Sustainability and environmental concern	Appeals to the sustainable nature of meat as a renewable source Appeals to meat not being sustainable or a threat to the environment Appeals to scale of meat farming industry	“Fish create less waste than other animals”; “Poor for environment”; “I think it is damaging to the environment to breed animals specifically for food”
Animal rights and welfare	Appeals to the death or suffering of animals entailed by meat eating Appeals to animal rights (i.e., animals have equal rights to life as humans)	“I don't like the idea of killing animals”; “It's wrong to harm animals”
Personal choice	Appeals to personal freedom of choice	“It's a personal thing”; “It is peoples own choice and personal references as to whether they eat meat or not”
Humane slaughter	Appeals to the “humane” nature of slaughtering practices	“As long as you know it comes from a company that does not mistreat animals”; “Humane options exist for meat products”

To examine our reasoning question, we divided our full sample based on their answer to the question “How okay or not okay is it to eat animals?” Participants who reported eating animals was “really not okay” (1) or “not okay” (2) formed the *opponents* group (*n* = 45, 15.7%). Participants who reported eating animals was “okay” (5) or “really okay” (6) were classified as *proponents* (*n* = 154, 53.7%). Participants who reported that eating animals was either “a bit not okay” (3) or “a bit okay” (4) were categorized as *ambivalent* (*n* = 88, 30.7%). We examined differences in the use of categories as a function of these evaluation groups.

Further reasoning analyses concentrated on the differences in category use as a function of dual or single argument use. Here, dual arguments were defined as any instance where a participant made use of two potentially conflicting arguments in favor or against eating meat, for example, “Because we need meat in our diet to stay strong and healthy as it gives us nutrients that other things can't, although I believe that animals are still living beings and do not deserve to die for out benefit as it is wrong” (see Table 2 for the full framework). Among our full sample, 66% of participants (*n* = 183) used single arguments, while 34% of participants (*n* = 93) used dual arguments.

**TABLE 2:** Coding framework dual vs single argument use.

Category	Definition	Examples
Single	Justifications which use only positive or only negative appeals of eating meat or animal products	“It's a life that doesn't need to be taken”; “Meat industry is bad for the environment”; “It's a person's choice so it's okay”; “it's the circle of life”; “I think it's okay because I sometimes eat meat when I can buy it cheaply. I rarely think about the animal that has been killed”
Dual	Justifications which use both positive and negative appeals simultaneously, often identifiable through phrasing including “but,” “however,” “while” Justifications which use phrasing such as “under specific circumstances…”; “if the circumstances…,” “as long as…” as this implies the participant has thought about both positive and negative aspects	“It can cause environmental damage but can also be done in a way that minimizes suffering to animals. It's also an ongoing food source, they keep producing eggs or milk for a period of time rather than killing the animal for its meat”; “Depends on circumstance a bit—again they suffer so it is worth trying to avoid”; “I think people should have a choice but also be made aware of the impact of eating meat”; “I think we should minimize but maybe not completely stop eating meat”; “I think it's fine to eat them as long as you do it sustainably. I personally don't eat animals”; “OK as long as the animals aren't harmed—which is very often the case”

To test our research questions, we first examined whether there were age‐related differences in membership of the evaluation groups (proponent, ambivalent, opponent) as a function of age group using chi‐square (RQ1) before testing for differences in speciesism as a function of age and evaluation group using a linear regression (RQ2). Following this, we used multinomial logistic regression to analyze reasoning category use as a function of age group and evaluation group (RQ3). This was followed by a multinomial logistic regression to examine reasoning category use as a function of evaluation group and argument type (RQ4). In these models, we included gender as a covariate given previous work with children that has done so (McGuire, Palmer, and Faber 2023) and work documenting the relation between gender and (evaluation of) meat eating (Salmen and Dhont 2023). In all analyses, the inclusion or exclusion of gender from the model did not change the model fit or likelihood ratio test results. All analyses were carried out in Jamovi (v.2.5, The Jamovi Project 2024).

## 3 Results

### 3.1 Demographics

First, we examined the demographics of opponents (*M* ^Age^ = 24.4, SD = 18.8, female *n* = 29, male *n* = 16), the ambivalent group (*M* ^Age^ = 20.6, SD = 15.9, female *n* = 53, male *n* = 35), and proponents (*M* ^Age^ = 26.2, SD = 19.2, female *n* = 56, male *n* = 98). In terms of dietary groups, among proponents of eating meat, 105 participants were omnivores, 6 were flexitarian, 2 were pescatarian, 2 were vegetarian, 10 followed other diets, and 29 did not report their diet. Among opponents of eating meat, 9 were vegetarians, 9 were vegans, 7 were omnivores, 2 were flexitarian, and 18 did not report their diet. Among the ambivalent group, 37 were omnivores, 10 were flexitarians, 2 were pescatarians, 1 was vegetarian, 1 followed another diet, and 37 did not report their diet.

### 3.2 Age and Evaluation

In a test of RQ1, a chi‐square contingency test demonstrated that the likelihood of belonging to the ambivalent, proponent, or opponent group depended on age group, *X* ^*2*^(4) = 18.6, *p* < 0.001, Cramer's V = 0.18. Among children, 40.8% (*n* = 40) were ambivalent, 38.8% (*n* = 38) were proponents, and 20.4% (*n* = 20) were opponents of eating meat. Among adolescents, 69.7% (*n* = 53) were proponents, 23.7% (*n* = 18) were ambivalent, and 6.6% (*n* = 5) were opponents of eating meat. Among adults, 55.8% (*n* = 63) were proponents, 26.5% (*n* = 30) were ambivalent, and 17.7% (*n* = 20) were opponents of eating meat.

### 3.3 Speciesism

To test for the relation between age and evaluation group on speciesism (RQ2), we conducted a linear regression with gender as a covariate. At the first step, we included the main effects of age group, evaluation group, and gender (adjusted *R* ^2^ = 0.22, AIC = 569, BIC = 593, *F*(5, 196) = 12.74, *p* < 0.001). The inclusion of an interaction between age group and evaluation group at the second step did not improve the fit of the model (adjusted *R* ^2^ = 0.22, AIC = 575, BIC = 612, *F*(9, 192) = 7.25, *p* < 0.001), and therefore, we focus on the main effects only. There was a main effect of evaluation group on speciesism (*F*(2, 196) = 13.58, *p* < 0.001). Relative to opponents, being a proponent positively predicted speciesism (*B* = 0.98, SE = 0.21, *t* = 4.57, *p* < 0.001), whereas there was no relationship between being in the ambivalent group and speciesism (*B* = 0.38, SE = 0.23, *t* = 1.61, *p* = 0.109). Hence, proponents of eating animals were more speciesist than opponents.

There was also a main effect of age group (*F*(2, 196) = 6.33, *p* = 0.002). Relative to children, there was a positive relationship between being in the adolescent group and speciesism (*B* = 0.62, SE = 0.29, *t* = 2.12, *p* = 0.04), whereas there was no relationship between being in the adult group and speciesism (*B* = 0.13, SE = 0.29, *t* = 0.45, *p* = 0.654). Compared to adolescents, there was a negative relationship between being in the adult group and speciesism (*B* = −0.49, SE = 0.15, *t* = −3.36, *p* = < 0.001). Hence, children and adults showed no significant differences in speciesism, while adolescents scored higher than both children and adults. Finally, there was a main effect of gender (*F*(2, 196) = 13.58, *p* < 0.001). Compared to female participants, there was a positive relationship between being male and speciesism (*B* = 0.38, SE = 0.14, *t* = 2.66, *p* = 0.008). This means that the relation between gender and speciesism demonstrated in adults (Caviola, Everett, and Faber 2019) also holds at a younger age.

### 3.4 Reasoning—Age and Evaluation

To examine differences in participants' use of reasoning categories as a function of age group and evaluation (RQ3), we conducted a multinominal logistic regression with use (1 = used, 0 = not used), evaluation group (proponents, ambivalent, opponents), age group (children, adolescents, adults), and gender (male, female) as a covariate. In Model 1, we included the main effects of use, age group, and evaluation group. In Model 2, we included the two‐way interactions between use and age, use, and evaluation group, and in Model 3, added the three‐way interaction between use, age group, and evaluation group. Model 2 was a better fit for the data than Model 1, and adding the three‐way interaction did not significantly improve the fit as indicated by an increase in AIC and BIC and no increase in *R* ^2^ (Model 1: AIC = 6104, BIC = 6295, *R* ^2^ = 0.01, *X* ^2^ (30) = 50.40, *p* = 0.011; Model 2: AIC = 5917, BIC = 6216, *R* ^2^ = 0.05, *X* ^2^ (50) = 277.80, *p* < 0.001; Model 3: AIC = 5697, BIC = 6483, *R* ^2^ = 0.05, *X* ^2^ (90) = 308.00, *p* < 0.001).

In interpreting the findings of our models, we used *animal welfare* as the reference category given that this is the main moral justification for not eating animals and to test what factors increase the likelihood of using other social‐conventional or personal categories. Therefore, in the following section, all references to likelihood refer to likelihood of using a reasoning category *relative to* the *animal welfare* category. Focusing on the model with the best fit (Model 2), likelihood ratio tests indicated that there was a significant interaction between use and evaluation group (*X* ^2^(10) = 133.78, *p* < 0.001). Coefficients (see Table 3) indicated that individuals who were proponents or ambivalent were more likely to refer to *humane slaughter*, *natural*, *normal*, *necessary*, and *nice* categories than opponents. In addition, proponents were more likely to refer to *humane slaughter*, *natural*, *normal*, *necessary*, and *nice* categories than those in the ambivalent group.

**TABLE 3:** Reasoning category use as a function of evaluation group.

	Predictor	Coefficient	Standard error	*Z*	*p*	Odds ratio	LLCI	ULCI
Humane slaughter	Use (1,0) * Evaluation Group (Ambivalent—Opponents)	2.49	0.76	3.26	0.001	12.07	2.7	53.93
	Use (1,0) * Evaluation Group (Proponents—Opponents)	4.27	0.78	5.46	< 0.001	71.8	15.47	333.28
	Use (1,0) * Evaluation Group (Proponents—Ambivalent)	1.78	0.62	2.87	0.004	5.95	1.76	20.07
Natural	Use (1,0) * Evaluation Group (Ambivalent—Opponents)	15.74	0.34	46.81	< 0.001	6.83E+06	3.53E+06	1.32E+07
	Use (1,0) * Evaluation Group (Proponents—Opponents)	18.63	0.33	56.34	< 0.001	1.24E+08	6.47E+07	2.37E+08
	Use (1,0) * Evaluation Group (Proponents—Ambivalent)	2.9	0.58	5.01	< 0.001	18.12	5.83	56.3
Necessary	Use (1,0) * Evaluation Group (Ambivalent—Opponents)	2.26	0.66	3.43	< 0.001	9.61	2.63	35.08
	Use (1,0) * Evaluation Group (Proponents—Opponents)	4.39	0.69	6.35	< 0.001	80.33	20.75	310.96
	Use (1,0) * Evaluation Group (Proponents—Ambivalent)	2.12	0.53	3.99	< 0.001	8.36	2.94	23.73
Nice	Use (1,0) * Evaluation Group (Ambivalent—Opponents)	2.4	0.95	2.52	0.012	22.01	1.71	70.98
	Use (1,0) * Evaluation Group (Proponents—Opponents)	5.33	0.93	5.74	< 0.001	205.41	33.36	1264.71
	Use (1,0) * Evaluation Group (Proponents—Ambivalent)	2.93	0.62	4.76	< 0.001	18.65	5.59	62.23
Normal	Use (1,0) * Evaluation Group (Ambivalent—Opponents)	15.21	0.39	38.82	< 0.001	4.03E+06	1.87E+06	8.69E+06
	Use (1,0) * Evaluation Group (Proponents—Opponents)	17.91	0.36	49.58	< 0.001	5.99E+07	2.95E+07	1.22E+08
	Use (1,0) * Evaluation Group (Proponents—Ambivalent)	2.7	0.63	4.27	< 0.001	14.87	4.31	51.28

*Note:* Animal Welfare is used as the reference category for all coefficients presented in Table 4.

Likelihood ratio tests indicated that there was also a significant interaction between use and age group (*X* ^2^(10) = 79.66, *p* < 0.001). Coefficients (see Table 4) indicated that adolescents and adults were more likely than children to refer to *humane slaughter*, *natural*, *normal*, and *necessary* categories. There were no age‐related differences in the likelihood of using the *nice* category. In addition, adults were more likely to use the *humane slaughter* category, while there were no differences between adults' and adolescents' likelihood of using the other categories.

**TABLE 4:** Reasoning category use as a function of age group.

	Predictor	Coefficient	Standard error	*Z*	*p*	Odds ratio	LLCI	ULCI
Humane slaughter	Use (1,0) * Age (Adolescents–Children)	3.09	0.94	3.29	0.001	21.91	3.48	138.05
	Use (1,0) * Age (Adults–Children)	4.53	0.87	5.24	< 0.001	71.80	15.47	333.27
	Use (1,0) * Age (Adults–Adolescents)	1.45	0.70	2.07	0.04	4.25	1.08	16.66
Natural	Use (1,0) * Age (Adolescents–Children)	3.49	0.72	4.86	< 0.001	32.85	8.03	134.45
	Use (1,0) * Age (Adults–Children)	3.81	0.69	5.53	< 0.001	45.12	11.69	174.11
	Use (1,0) * Age (Adults–Adolescents)	0.32	0.64	0.49	0.621	1.37	0.39	4.83
Necessary	Use (1,0) * Age (Adolescents–Children)	1.83	0.60	3.07	0.002	6.26	1.94	20.20
	Use (1,0) * Age (Adults–Children)	2.50	0.54	4.62	< 0.001	12.17	4.21	35.16
	Use (1,0) * Age (Adults–Adolescents)	0.66	0.62	1.07	0.285	1.94	0.56	6.57
Nice	Use (1,0) * Age (Adolescents–Children)	1.14	0.66	1.73	0.084	3.14	0.86	11.51
	Use (1,0) * Age (Adults–Children)	1.07	0.64	1.67	0.094	2.91	0.83	10.15
	Use (1,0) * Age (Adults–Adolescents)	−0.08	0.73	−0.11	0.916	0.93	0.22	3.86
Normal	Use (1,0) * Age (Adolescents–Children)	1.44	0.76	1.88	0.06	4.21	0.94	18.78
	Use (1,0) * Age (Adults–Children)	2.76	0.66	4.16	< 0.001	15.84	4.31	58.23
	Use (1,0) * Age (Adults–Adolescents)	1.33	0.75	1.78	0.075	3.76	0.87	16.20

*Note:* Animal Welfare is used as the reference category for all coefficients presented in Table 4.

### 3.5 Reasoning—Evaluation and Argument Type

To examine differences in participants' use of reasoning categories as a function of argument type and evaluation (RQ4), we conducted a multinominal logistic regression with use (1 = used, 0 = not used), evaluation group (proponents, ambivalent, opponents), argument type (dual, single), and gender (male, female) as a covariate. In Model 1, we included the main effects of use, argument type, and evaluation group. In Model 2, we included the two‐way interactions between use and argument type and between use and evaluation group. In Model 3, we included the three‐way interaction between use, argument type, and evaluation group. Model fit measures suggested that Model 2 was the best fit for the data (Model 1: AIC = 5941, BIC = 6104, *R* ^2^ = 0.01, *X* ^2^ (25) = 52.80, *p* < 0.001; Model 2: AIC = 5784, BIC = 6027, *R* ^2^ = 0.04, *X* ^2^ (40) = 240.60, *p* < 0.001; Model 3: AIC = 5805, BIC = 6157, *R* ^2^ = 0.04, *X* ^2^ (60) = 259.60, *p* < 0.001).

Again, in interpreting the findings of our models, we used *animal welfare* as the reference category and the following statements refer to likelihood of using a reasoning category *relative to* the *animal welfare* category. Likelihood ratio tests indicated that both the two‐way interaction between use and evaluation group (*X* ^2^(10) = 147.72, *p* < 0.001) and use and argument type (*X* ^2^(5) = 39.69, *p* < 0.001) were significant. As we have discussed the reasoning category use and evaluation group interaction above, here we focus on the reasoning category use and argument type interaction (see Table 5 for coefficients). Participants who used dual arguments were more likely than those who used a single argument to refer to the *humane slaughter*, *nice*, and *necessary* categories. There was no difference between dual argument and single argument groups in the likelihood of using the *natural* or *normal* categories.

**TABLE 5:** Reasoning category use as a function of argument type.

	Predictor	Coefficient	Standard error	*Z*	*p*	Odds ratio	LLCI	ULCI
Humane Slaughter	Use (1, 0) * Argument Type (Dual–Single)	1.72	0.52	3.32	< 0.001	5.58	2.02	15.41
Natural	Use (1, 0) * Argument Type (Dual–Single)	−0.23	0.47	−0.48	0.63	0.78	0.32	2.00
Necessary	Use (1, 0) * Argument Type (Dual–Single)	1.94	0.45	4.35	< 0.001	6.98	2.90	16.76
Nice	Use (1, 0) * Argument Type (Dual–Single)	1.06	0.51	2.06	0.039	2.89	1.05	7.90
Normal	Use (1, 0) * Argument Type (Dual–Single)	0.77	0.53	1.46	0.144	2.17	0.77	6.13

*Note:* Animal Welfare is used as the reference category for all coefficients presented in Table 5.

## 4 Discussion

In the present study, we sought to understand the forms of evaluation and reasoning used by children, adolescents, and adults when asked about eating animals. Specifically, guided by social domain theory (Turiel 1983) and research on the 4 N's (Piazza et al. 2015), we aimed to extend research in this area by understanding the reasoning of proponents, ambivalent reasoners, and opponents of meat eating across three age groups, as well as the role of single versus dual argument use. There were differences in endorsement for eating animals based on age. Adults and adolescents were more likely than children to be proponents of eating meat. In contrast, children were more likely to be ambivalent about eating meat. There were differences in reasoning by evaluation group, age group, and argument type. Being in the proponent or ambivalent group increased the likelihood of using the 4Ns (e.g., *natural*) and personal domain (e.g., *nice*) compared to moral domain (e.g., *animal welfare*) reasons. Being an adult or adolescent increased the likelihood of referring to *humane slaughter* practices, as well as categories of *natural*, *normal*, and *necessary*. Finally, individuals who used dual arguments were more likely to refer to *humane slaughter*, the *necessity* of eating meat, and meat eating as *nice*.

Together this evidence reveals a developmental picture where both the age of the individual *and* their attitude toward meat eating determine the kinds of reasoning that are used. Increasingly with age we see reference to social conventions, biological justifications of meat eating as necessary or natural, and personal domain reasoning in contrast to animal welfare. This is consistent with existing empirical work with adults (Piazza et al. 2015) and with children (McGuire et al. 2023), as well as with theoretical accounts (Richardson, Mulvey, and Killen 2012) regarding the coordination and increasing importance of social conventions with age. What distinguishes the concept of meat eating from other reasoning contexts previously studied within the social domain theory framework is the shift with age for some people from seeing a topic as morally relevant to being seen as belonging to the conventional domain or with appeal to biological necessity. In other contexts, conventions come to bare on moral domain issues with age. For example, while people generally endorse fairness as a moral principle, increasingly with age individuals will argue that there are social conventions, such as group norms, that may mean fair distribution of resources is not warranted (McGuire et al. 2018). What marks meat eating as unique is that some individuals seem to move away from seeing meat eating as defined by the moral domain and instead are more likely to refer to social conventions not *as a means to* navigate a moral situation, but instead see meat eating *itself* as conventional.

From the social domain theory perspective, social‐conventional domain issues are distinguished by a degree of normativity (i.e., a convention is acceptable in part because it is agreed upon by members of that context). When it comes to meat eating, individuals refer to conventions that pitch eating meat as *normal* (i.e., many people do it) and biological or evolutionary justifications of meat eating as *natural* (i.e., as a species we have always done it) rather than as a moral domain harm issue that is qualified by conventions. As children and adolescents come to understand that adherence to conventions is particularly important to inclusion within groups (Abrams et al. 2003; Mulvey et al. 2014; Travaglino et al. 2014), the shift to seeing humans' use of animals as food as an important convention could play a central role in age‐related changes in how animal lives are valued.

In the present work, we set out the argument that social domain theory is a valuable framework for understanding children's emerging reasoning about eating meat. We argue that this model is helpful for understanding the conflict between moral domain issues (harm to animals), social conventions (“it is normal to eat meat”), and personal concerns (“meat eating is my own choice to make”). Consistent with the suggestion that children utilize these domains of knowledge, we saw references to all three domains, sometimes in conjunction with one another. However, it is important to note that also other reasons were cited by participants—most prominently, biological and evolutionarily based arguments for meat eating as being natural and necessary to human life. Such arguments do not fit neatly within the three domains of social domain theory. Rather, they appear to rely on ideas that serve to create a greater psychological distance between humans and nonhuman animals (Bastian, Costello, et al. 2012) by reinforcing the idea that the use of animals for food is determined by evolution and essential to our species' survival. Social domain theory, while an influential theory of moral development, by design does not assess such beliefs. While we here used social domain theory as a grounding theory due to its focus on reasoning and our focus on the moral standing of animals, future work in this area will require a broader theoretical focus to fully capture the breadth of developing thinking about nonhuman animals.

Outside of social domain theory, the present findings have connections to other lines of theory in the human–animal intergroup relations literature. Researchers have examined the possibility that reasoning is one means by which the conflict between caring for animals and eating animals can be resolved (Piazza et al. 2015). This conflict has been theorized to relate to meat‐related cognitive dissonance (Rothgerber 2020a, 2020b; Rothgerber and Rosenfeld 2021). Theorists have argued that in the context of meat, dissonance arises when an individual holds the belief that causing harm to other living beings is wrong, while at the same time practicing an omnivorous diet in the knowledge that this diet causes harm to animals (Rothgerber 2020a).

From a related developmental perspective, a recent model put forward by Piazza, Simpson, and McGuire (2023) argues that it is this conflict between an early moral concern for animals and an *emerging* knowledge about how food is produced that leads to a cascade of psychological corollaries in support of meat eating. The authors argue that in childhood this conflict is likely to be low due to a lack of knowledge of food systems processes (Bray et al. 2016; Hahn, Gillogly, and Bradford 2021). In other words, children do not have the motivation nor need to use certain categories of reasoning (e.g., references to humane slaughter) to overcome dissonance, as they simply do not grasp the suffering involved in meat production. In adolescence, the authors suggest that this conflict is likely to emerge as more comprehensive and accurate knowledge of food systems develops. To reduce dissonance, adolescents have the option to either reduce their meat consumption or to turn to “adult” like psychological mechanisms, including reasoning. The emergence of this dissonance between childhood and adolescence may then relate to differences in belonging to different evaluation groups and in turn, the reasoning they use.

Interestingly in the present work, we observed that 40% of 9‐ to 11‐year‐old children were ambivalent about eating animals (i.e., either stated that eating animals was “a bit okay” or “a bit not okay”). This finding puts in context existing work on children's moral judgments about animals (McGuire et al. 2023; Wilks et al. 2021) by demonstrating that while in general terms children are less likely to prioritize humans over animals and more likely to think about animal welfare compared to adults, there are children who are less sure of how to evaluate *eating* animals. One possibility is that it is among this group that we first see some of the emerging conflict between concern for animals and understanding of how many animals are treated.

In line with the possibility that there are differences in childhood regarding awareness of how meat is produced, recent research has shown that young children (5–8 years old) make errors in recognizing where meat comes from (Hahn, Gillogly, and Bradford 2021) and parents are reluctant to talk to their children about where their food comes from (Bray et al. 2016). The emergence of the *humane slaughter* category seen in the present work likely relies on an understanding of factory farming practices combined with a continued omnivorous diet in adolescence and adulthood. This category includes references to the idea that there may be more humane or ethical ways to rear and kill animals, compared to factory farming. Use of the *humane slaughter* category was higher among the ambivalent and proponent groups, as well as among adults and adolescents. For example, one adult participant stated: “*I believe that it can be ok to eat meat in certain circumstances provided the animals are raised and slaughtered in an ethical manner*.” Future research that explicitly seeks to understand when this knowledge first emerges will be an important next step. Making connections between reasoning and related psychological concepts in the human–animal intergroup relations literature will also be an important step. For example, understanding how this knowledge about the origins of food relates to the categorization of different species will shed further light on developing attitudes to nonhuman animals. Related work has documented that with age, farmed animals are more likely to be categorized as “food” than “pet” (McGuire, Palmer, and Faber 2023). It is likely that these findings represent a suite of related processes that lead to a more “adult” view of animals (Loughnan, Bastian, and Haslam 2014). Understanding the reality of human–animal intergroup relations may necessitate a shift toward more hierarchical thinking, changing categories, and more conventional reasoning regarding animals.

Another novel finding of the present work is the focus on individuals who reference conflicting positions in dual arguments. For those who rely on these dual arguments, concern for animal welfare is expressed through references to harmful practices involved in animal agriculture. Yet, at the same time, this group weighs these concerns up against other issues, for example, through stating that while eating animals has unfortunate ethical consequences, it is necessary to eat animal protein to promote human interests. For example, one adult participant stated that it was okay to eat animals because “they provide food for our population, but the way we kill and look after the animals needs to improve.” Even when the individual explicitly recognizes that they personally disagree with harming animals, in the same response participants can counter this moral principle with a reference to dietary necessity for personal wellbeing. Again, we see reference to the utility of animals as food underpinning a conflict between morality and convention and a possible contribution of reasoning toward the resolution of this conflict. Future research following up with these participants who engage in dual arguments to understand whether there are “profiles” of individuals more likely to directly reference this conflict will be a valuable contribution to the literature in this area. For example, in the present work, we did not observe differences in the use of single and dual arguments as a function of the participants' evaluation, suggesting that dual reasoning was apparent among proponents, opponents, and the ambivalent group. This reflects the complexity that individuals seem to face when reasoning about meat consumption. Even participants who are strongly opposed or in favor of meat eating highlight potentially conflicting statements. If evaluation itself is not a strong predictor of single and dual reasoning, an important future direction will be understanding the individual differences or contextual factors that might predict whether one is likely to use such arguments and in turn the consequences of single versus dual reasoning for future eating decisions.

In addition, we were not able to probe the relationship between age, argument type, meat‐eating evaluation, and reasoning category simultaneously in this study. To investigate this complex relationship would likely require a substantial sample for quantitative analyses, plus in‐depth qualitative research to further understand how individuals of different ages reconcile the conflict between competing arguments. Despite this, it is of note that in the present study participants of all ages engaged in dual reasoning. This suggests that the internal conflict between arguments about eating animals can be present in childhood and further emphasizes the importance of understanding who this group comprises and what mechanism sparks this initial conflict.

These findings suggest important implications for encouraging the uptake of environmentally sustainable and healthy diets. In childhood, there is a focus on animal welfare relative to adolescents who begin to stress the importance of conventions, biology, and humane slaughter practices. These findings suggest that there is scope for different kinds of argument depending on the age of the individual. In adolescence, attempts to advocate for sustainable diets will need to proceed cautiously, recognizing the power of conventions and offering opportunities to reduce the unpleasant dissonance that may arise when discussing how food is produced. In addition, from a lifespan development perspective, there remains an ambivalent group within the adult population who raise the issue of animal welfare as part of their *humane slaughter* reasoning. Work which seeks to understand who this group are and the types of messages that may lead to a shift from ambivalence to either being a proponent or opponent of meat eating will be a valuable next step for researchers interested in the psychology of meat.

### 4.1 Limitations and Future Directions

The present study sampled cross‐sectionally from across the lifespan, which limits our ability to make strong claims regarding the development of reasoning. To complement this initial investigation, longitudinal research will be essential to examine how, for example, the emergence of food systems knowledge is related to changes in the use of reasoning categories within individuals across development. In addition, we sampled from 9 years old, meaning that our data cannot tell us about reasoning processes in early childhood. Given work in this area has documented that 5‐ to 9‐year‐old children in the United States and Poland are less likely to prioritize human lives over animal lives (Paruzel‐Czachura et al. 2024; Wilks et al. 2021), it will be particularly interesting to further understand how the kinds of reasoning we have seen in the present work manifest in younger children. One might expect given such findings and the age‐related decrease in use of *animal welfare* reasoning, younger children would be even more likely to refer to animal welfare and rights. Future work is essential to probe this possibility.

In addition, our analyses revealed that male participants were likely to score higher than female participants on a standardized speciesism measure. This suggests that from a young age there are potential differences in thinking about animals as a function of gender, in line with recent theorizing (Salmen and Dhont 2023). Understanding the age at which these differences emerge and how these differences may relate to ways in which boys and girls are socialized around food will be a valuable future direction for work in this area.

Further, we recognize that there are limitations in relying solely on participants' explicit post hoc justifications for meat‐eating behavior, given that implicit attitudes make independent contributions to behavior in childhood (Cvencek, Greenwald, and Meltzoff 2011) and the processes involved in upholding meat eating often take place outside of the immediate awareness of the individual (Bastian, Loughnan, et al. 2012). Again, future work ought to consider the use of both explicit and implicit measures, as well as behavioral approaches to fully capture the development of the psychology of meat eating.

Importantly, data were collected in one cultural context only, from a majority White British sample. Food practices and production are highly culturally specific, as are judgments about the moral value of animals (Tian, Hilton, and Becker 2016; Tian et al. 2020). As a result, it seems highly likely that age‐related reasoning will differ as a function of culture. For example, in cultures where food production is less hidden and euphemistic compared to the United Kingdom, it may be that speciesist thinking and reasoning emerge earlier in development. Even within the United Kingdom, there are children with different experiences with animals (e.g., children of farmers) who likely reason differently about whether it is acceptable to eat animals. Given this caveat, we urge caution in extrapolating too far beyond our sample of majority White British participants and encourage future replications. Future cross‐cultural work will be a highly important next step before strong claims about the development of human–animal intergroup relations can be made.

Finally, while the current work did focus on dual arguments, due to the many combinations of category use we were not able to statistically examine all the different iterations of conflicting arguments, nor to examine how age, evaluation, and dual versus single argument use crossover. A qualitative examination of ambivalent participants' reasoning that further probes which categories are most often in conflict and why will be a valuable addition to the literature.

### 4.2 Conclusion

Understanding human–animal intergroup relations across the lifespan has important applications in efforts to increase the uptake of plant‐based diets, improve health outcomes, and advance social equality for animals and humans alike (e.g., since speciesism is itself linked to prejudice toward other humans; Caviola, Everett, and Faber 2019). Central to this psychology is the conflict between humans' inherent concern for living beings and the harm caused by animal agriculture. This conflict is in part resolved by adults through reasoning, which in the present work appears to begin to increasingly focus on “natural” hierarchies and social conventions between childhood and adolescence. It is, however, not the case that all individuals move from animal welfare concern to unambivalent meat‐eating endorsement, as our study reveals a portion of ambivalent individuals who engage with dual arguments directly referencing a conflict between concern for animal lives and conventions around food. For advocates interested in advancing arguments regarding animal equality, countering the position of proponents will be difficult. In contrast, persuading ambivalent adolescents and adults might be possible, though it will take careful messaging to complement the concern for animal lives seen in the humane slaughter arguments in the current work. Efforts focused on encouraging the uptake of plant‐based diet for planetary health would do well to examine this ambivalent group in more depth across the lifespan.

## Data Availability

The authors have nothing to report.
